# Maternal nutrition counselling is associated with reduced stunting prevalence and improved feeding practices in early childhood: a post-program comparison study

**DOI:** 10.1186/s12937-019-0473-z

**Published:** 2019-08-27

**Authors:** Sabuj Kanti Mistry, Md. Belal Hossain, Amit Arora

**Affiliations:** 1 BRAC James P Grant School of Public Health, BRAC University, 68 Shahid Tajuddin Ahmed Sharani, Mohakhali, Dhaka, 1212 Bangladesh; 2 Centre for Primary Health Care and Equity, University of New South Wales, Sydney, Australia; 3 School of Science and Health, Western Sydney University Campbelltown Campus, Locked Bag 1797, Penrith, NSW 2751 Australia; 4 Translational Health Research Institute, Western Sydney University, Locked Bag 1797, Penrith, NSW 2751 Australia; 5 Discipline of Child and Adolescent Health, Sydney Medical School, Faculty of Medicine and Health, The University of Sydney, Westmead, NSW 2145 Australia; 6 Oral Health Services, Sydney Local Health District and Sydney Dental Hospital, NSW Health, Surry Hills, NSW 2010 Australia

**Keywords:** Stunting, Nutrition counselling, Community health worker (CHW)

## Abstract

**Background:**

Despite progress, suboptimal feeding practices and undernutrition particularly in the form of stunting still remains a major issue among children aged less than 5 years in Bangladesh. Since mothers are the primary caregivers of young children, maternal nutrition counselling can be effective in improving knowledge and practices on child feeding. The Building Resources Across Communities (BRAC) initiated a nutrition counselling intervention using its essential health care (EHC) skeleton in 114 sub-districts of Bangladesh in 2012. This study assessed the role of this intervention on the prevalence of stunting and feeding practices among children aged less than 5 years.

**Methods:**

The data was collected as part of a nationwide cross-sectional survey, which followed a two-stage cluster random sampling procedure and was conducted between October 2015 and January 2016. The present study analyzed the information of 3009 mother-children dyads from two selected survey areas: i) areas where the EHC package was delivered (comparison; *n* = 1452), ii) areas with EHC plus nutrition counselling package (intervention; *n* = 1557) was delivered. The Chi-square test was done to compare the child feeding practices and stunting prevalence between intervention and comparison. The degree of strength of the association of stunting and the intervention was estimated using a mixed-effect logistic regression model.

**Results:**

The study revealed that the prevalence of stunting was significantly lower in areas where the intervention was delivered compared to the comparison areas (29% vs. 37%, *P <* 0.001). Furthermore, after adjusting for administrative zone, household wealth quintile, child’s age, gender, maternal age, education, occupation, cluster disparity, and variation between study groups, it was seen that the risk of stunting was 25% lower in the intervention areas compared to the comparison areas (aOR: 0.75, 95% CI: 0.60–0.94; *P =* 0.012). Optimal child feeding practices were also more common among mothers from intervention areas than those of the comparison areas (exclusive breastfeeding: 72.7 vs. 59.4%, *P* = 0.008; feeding 4+ food groups: 42.9 vs. 34.1%, *P* < 0.001; having minimum acceptable diet: 31.2 vs. 25.3%, *P* = 0.017; feeding multiple micro-nutrient powder: 16.2 vs. 7.4%, *P* < 0.001).

**Conclusions:**

The study highlighted that nutrition counselling of mothers may be effective in reducing childhood stunting with concomitant improvement in optimal feeding practices in children under 5 years of age. The frontline community health workers can be trained to counsel mothers on optimal child feeding practices and this could help reduce the prevalence of stunting.

## Background

Globally, 3.1 million children under the age of five years (under-5) die annually due to undernutrition, which is nearly half of all the deaths occurring in children under-5 [1, 2]. The World Health Organization (WHO) highlights stunting is the most predominant form of undernutrition among children under-5 and it is estimated that globally around 161 million under-5 are stunted [3].

Although childhood stunting is ubiquitous round the world, it is particularly prevalent in low- and middle-income countries (LMICs) [4, 5]. According to the most recent Demographic and Health Survey, more than one-third (36.1%) of under-5 children are stunted in Bangladesh [6]. Despite this, Bangladesh is one of the few LMICs which has achieved a considerable reduction in the prevalence of childhood stunting over the last decade [4, 6]. This progress can heavily be attributed to the collaborative efforts of the Government of Bangladesh, a few development partners, and non-government organizations (NGOs) in implementing several community based healthcare and nutrition interventions [7].

Despite considerable reduction in the prevalence of childhood stunting over the last decade, the reduction rates are relatively slow in Bangladesh [6]. A recent study affirmed that the annual average reduction in the prevalence of stunting is 2.9% in Bangladesh [8] and with this pace of reduction it is less likely to achieve the World Health Assembly’s target of reducing the prevalence of stunting by 40% before the year 2025 [9–11]. This implies that the government, policy makers, and researchers need to search for effective initiatives to sustain the progress and to accelerate the rate of stunting reduction.

A wealth of evidence generated in resource poor settings highlighted that nutrition education to mothers of young children could significantly increase the knowledge and improve practices on optimal early childhood feeding, which was significantly associated with the lower prevalence of undernutrition among their children [12–14]. In fact, suboptimal feeding practices in childhood has been identified as one the most three prominent drivers of childhood stunting in South Asian countries [15]. Although some studies carried out in resource poor setting reported that maternal nutrition counselling is associated with improved infant and young child feeding (IYCF) practices [16, 17] and reduced childhood stunting [18–20], there is limited evidence from Bangladesh.

In 2011, Building Resources Across Communities (BRAC) initiated an intervention in 114 sub-districts of Bangladesh where the community health workers (CHWs) of BRAC namely *Sasthya Kormis* (*SK*s) were trained to counsel mothers on childhood nutrition in addition to their primary role of providing basic healthcare services, which was in place since 1991. On the other hand, in 131 sub-districts, the *SK*s continued to deliver the basic healthcare services [21]. The purpose of this study was to assess the impact of this intervention on the prevalence of stunting and feeding practices among children aged less than 5 years.

## Methods

### Study design and participants

The data used for this study was part of a nationwide cross-sectional health care survey conducted by BRAC between October 2015 and January 2016. The study area was divided into 7 domains with different combinations of major health interventions of BRAC. Totally, it covered 457 sub-districts among 492 sub-districts of Bangladesh as well as the entire urban slums from all of the 11 city corporations of the country.

Essential health care (EHC) is the fundamental health initiative of BRAC which has been in operation since 1991 and currently covering 4.8 million population in 363 sub-districts of rural areas of Bangladesh. All other major health initiatives of BRAC has been established on this EHC backbone except in improving maternal, neonatal and child survival (IMNCS) areas where the EHC model has been restructured into IMNCS model. Thus, at the end of 2011, there were 245 sub-districts with EHC operation only, of which 114 sub-districts were randomly selected where the nutrition package was inserted into the EHC backbone in 2012. Therefore, we considered two domains in present study: i) areas with essential health care (EHC) package only (comparison), ii) areas with EHC package plus nutrition initiative (intervention).

**Fig. 1:**
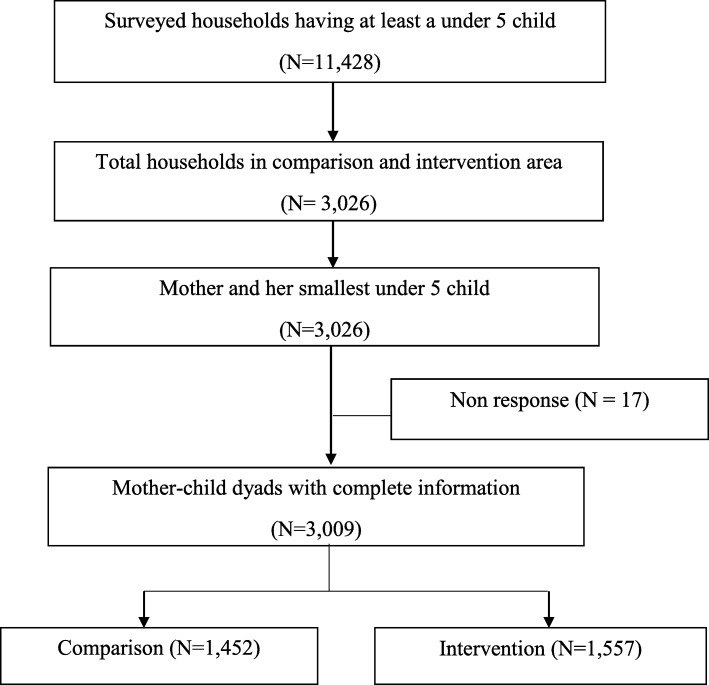
Study profile and participants enrollment

In the 2015–16 survey, a two-stage cluster random sampling procedure was applied and 11,428 households having at least one under-5 child were selected. In the first stage, 210 enumeration areas (EAs) i.e., 30 EAs from each of the 7 domains were selected randomly, with 180 EAs from rural areas and thirty EAs from urban slums. An EA is a union (rural areas) or ward (slums), which is the lowest administrative unit in Bangladesh. In the second stage, on an average, fifty-four households were selected per EA through systematic random sampling to provide statistically reliable estimates. If a household had more than one under-5 child, the youngest child was included in the survey. The details can be found elsewhere [22]. The present analysis was performed among 3009 mother-children dyads; 1452 sampled from comparison areas and 1557 from intervention areas. The detail sampling procedure is presented in Fig. 1.

### Overview of EHC and nutrition intervention of BRAC

EHC delivers low cost basic curative and preventive health care services, particularly focusing on mother and children. Employing frontline CHWs i.e., *Shasthya Sebikas* (*SSs*) and *Shasthya Karmis* (*SKs)*, EHC offers health services through door-to-door visits of the targeted beneficiaries. In fact, this CHW skeleton is the basis of health intervention of BRAC [21]. *SS*s are the grass-root CHWs of BRAC, recruited from the target community, married women aged between 25 and 40 years, and at-least primary level of education. They receive 15 days training on basic health care services as well as on health issues of mothers and children under-5, delivered by medical doctors and program personnel. They also join a monthly refresher training to help retain their knowledge and skills. Every *SS* has a working area of 200–300 households and are supervised by a *SK*, the second layer of CHWs. *SK*s roughly aged between 20 to 35 years with secondary education completed, and each *SK* has the responsibility of monitoring 10–12 *SS*s. *SKs* receive an intensive 18 days residential training provided by medical doctors and program personnel covering maternal and child health issues as well as on data reporting and delivering antenatal, postnatal, and child care services. Alike *SS*s, *SK*s also join monthly refresher training. Both of these CHWs provide treatments for basic ailments and performs tasks such as pregnancy identification, family planning services, antenatal care, assistance in child birth, neonatal and child care, and detection and referral for maternal and childhood complications [23, 24].

In 2012, BRAC initiated a nutrition intervention package in 114 EHC rural sub-districts using the CHWs of BRAC with an aim to improve the nutrition status of the under-5 children. The *SK*s’ in these areas were provided with an intensive training on child nutrition for 7 days. In addition to their regular EHC activities they also performed nutrition counselling targeting mothers of the children under-5 to promote optimal feeding practices and nutrition in children. *SK*s were asked to counsel mothers on proper nutrition during pregnancy, iron-folate and calcium supplementation during pregnancy and lactation, importance of breastfeeding, and age-specific complementary feeding in addition to the EHC activities of promotion of antenatal and postnatal health care services.

### Data collection tools and techniques

A pre-tested structured questionnaire was administered to collect the household, mother, and child related information through face-to-face interview of the mothers, while anthropometric measurements of the children were taken using standardized procedures. Collected information was recorded electronically in an android based mobile app, ODK (Open Data Kit), with an efficient user-friendly interface, which is particularly effective in low resource settings. ODK can be performed in both online and offline and can be administered by people with minimal educational attainment [25].

A total of 84 female research assistants with previous experience in conducting heath care survey were hired to conduct the interviews. Since our respondents were women, we preferred female interviewers to minimize reporting bias [26]. They were trained intensively for 15 days with a series of classroom lectures, role play, mock interviews, and field practice. A total of 21 groups were formed, each consisting of four research assistants led by a trained supervisor. Data collection was done in an offline version of ODK and was uploaded in evenings on a daily basis. The server was controlled by an ICT expert at the Dhaka Head Office. A strong monitoring system was set up in place and activities such as spot inspection, questionnaire scrutiny, and back checking was performed to validate and maintain data quality and necessary feedback was provided to the research assistants. The team leader acted as the first level of monitoring while researchers and field managers frequently travelled to the survey areas to supervise data collection. A statistician was also positioned in the Head Office to monitor the flow of data, quality of live data, and to provide immediate feedback to the respective interviewers.

### Measures

Construction of the wealth index was based on factor analysis [27, 28] of key socioeconomic variables. The key socioeconomic variables were: types of wall, floor and roof of the house, ownership of radio, television, computer, bicycle, mobile/telephone, refrigerator, wardrobe, table, chair, watch, bed, sewing machine, bike, motor vehicle, livestock, and access to solar, electricity.

Child’s age was recorded from the immunization card or in its unavailability, the local event calendar was used. Weight of the children was measured using electronic bathroom scale to the nearest 0.1 kg precision, while the height was measured by using a locally prepared board with a wooden base in supine position and a movable headpiece, on a flat subject with 0.1 cm precision. Anthropometric index height-for-age z-score was constructed using WHO Anthro Plus software [29].

Feeding practices were measured through interviewing mothers of the children aged less than two years because the WHO/UNICEF guidelines for IYCF practices should be recorded in children aged 0–23 months [30]. Pre-lacteal feeding was defined as children given any food except breast milk within first three days of life, while a child was considered to be exclusively breastfed if he/she did not receive anything except breast milk (even not a drop of water) in the 24 h preceding the survey [31]. Minimum dietary diversity was defined if children aged 6–23 months received foods from at least 4 out the 7 food groups in the last 24 h preceding the survey. Minimum meal frequency was defined as breastfed children 6–8 months received 2–3 meals per day, breastfed children 9–23 months received 3 or more meals per day, and non-breastfed children received 4 or more meals per day. The children were considered as having the access to minimum acceptable diet if they satisfied both the conditions of minimum dietary diversity and minimum meal frequency [32]. Meanwhile, children were considered micro-nutrient powder (MNP) fed if they were given one or more multiple MNP sachets and considered effectively MNP fed if given ≥60 sachets in last 6 months prior to the survey [33].

### Outcome variable

The primary outcome of this study was stunting. A child with less than two standard deviations below the median (− 2 SD) of the WHO reference population in terms of height-for-age z-score was considered as stunted [34].

### Statistical analysis

Descriptive analysis was performed to assess the distribution of variables. The Chi-square test was used for comparing maternal knowledge, practice, and childhood stunting between comparison and intervention areas with 5% level of significance. Since the data were nested in nature and there were cluster (union) to cluster variation and disparity between study groups, the mixed-effect logistic regression model by considering cluster variations and study groups as random effects was performed to assess the role of maternal nutrition counselling. The final model was adjusted with administrative area, household wealth quintile, child’s age, gender, maternal age, education and occupation. The adjusted odds ratio (aOR) and confidence interval (CI) were estimated by considering 5% level of significance. All analyses were carried out using the statistical software package STATA (Version 13.0).

## Results

### Background characteristics

Among 1452 children from comparison areas, 43.5% were from the central Bangladesh, 41.2% from southern Bangladesh, and 13.2% from the northern part of Bangladesh (Table 1). A total of 41.8% children were from poor households (20.7% poorest, 21.1% poorer). Only 9.2% children were below 6 months of age while 43.8 and 47.0% were between 6 and 23 months and 24–59 months of age, respectively. The male-female ratio was close to unity. Approximately two-third mothers aged between 20 and 29 years, and most of the mothers were housewives. Moreover, 6.8% mothers had no education and 20.9% had completed grade 10 or more.

**Table 1:** Background characteristics of study population

Characteristics	EHC		EHC + Nutrition	
	n	%	n	%
Administrative zone				
North	223	15.36	206	13.23
Central	631	43.46	541	34.75
South	598	41.18	810	52.02
Household wealth status				
Poorest	301	20.73	256	16.44
Poorer	307	21.14	280	17.98
Middle	273	18.80	346	22.22
Richer	307	21.14	386	24.79
Richest	264	18.18	289	18.56
Child age (months)				
0–5	133	9.16	256	16.44
6–23	636	43.80	718	46.11
24–59	683	47.04	583	37.44
Child gender				
Male	729	50.21	818	52.54
Female	723	49.79	739	47.46
Maternal age (years)				
< 20	126	8.68	221	14.19
20–29	936	64.46	955	61.34
≥ 30	390	26.86	381	24.47
Maternal education				
No schooling	98	6.75	87	5.59
Primary incomplete^a^	239	16.46	258	16.57
Primary or secondary incomplete^b^	812	55.92	894	57.42
Secondary or higher^c^	303	20.87	318	20.42
Maternal occupation				
Housewife	1380	95.04	1487	95.50
Working outside	72	4.96	70	4.50
N	1452	100.0	1557	100.0

^a^Completing grade 1–4, ^b^completing grade 5–9, ^c^completing grade 10 or higher

Of the 1557 children from intervention areas, 34.8% resided in central Bangladesh, while 52.0% were from southern Bangladesh, and 13.2% from the northern part of Bangladesh (Table 1). A total of 34.4% children were from poor households (16.4% poorest, 18.0% poorer). Approximately 16.4% children were less than 6 months of age, and nearly 50% aged between 6 and 23 months. The male-female ratio was also close to unity. Moreover, 61.3% mothers were aged between 20 and 29 years, and the percentage of educated mothers and housewives were nearly similar to those in comparison areas (Table 1).

### Maternal knowledge on child feeding following intervention

Although 8.6% mothers in comparison areas had knowledge on prelacteal feeding, it was significantly lower in the intervention areas (4.1%) (Table 2). The knowledge on initiation of breastfeeding within the first hour of birth was also significantly higher among mothers from the intervention areas than comparison areas (89.5% vs. 84.8%, *P* = 0.003). Although the percentage of mothers having knowledge on exclusive breastfeeding up to 6 months in both areas were similar, knowledge on initiation of complementary feeding by 7 months was significantly higher in intervention areas (93.3% vs. 90.3%, *P* = 0.019). Moreover, mothers from intervention areas had significantly higher knowledge on dietary diversity for children, i.e., having 4 or more food groups in a day, than comparison areas (86.8% vs. 80.0%, *P* < 0.001) (Table 2).

**Table 2:** Maternal knowledge on child feeding in comparison and intervention area

Characteristics	Comparison area	Intervention area	*P* ^1^
Number of children aged < 2 years (N)	769	974	
Prelacteal feeding (%)	8.58	4.11	< 0.001
Initiation of breastfeeding within 1 h of birth (%)	84.79	89.53	0.003
Exclusive breastfeeding up to 6 months (%)	89.99	91.99	0.144
Initiation of complementary feeding by 7 month (%)	90.25	93.33	0.019
Having 4+ food groups in a day (%)	79.97	86.76	< 0.001

^1^ *P*-value for the Chi-square test between comparison and intervention areas

### Maternal practices on child feeding following intervention

Although 13.3% mothers in comparison areas provided prelacteal feed to their child, it was significantly lower in the intervention areas (9.8%) (Table 3). Moreover, 72.7% women exclusively breastfed their children (aged less than 6 months) in the last 24-h before the survey in the intervention areas compared to 59.4% in comparison areas (*P* = 0.008). Although, among mothers with 6–23 months aged children, the practices of initiation of complementary feeding by 7 months was nearly similar in both areas (69.1 and 70.1% respectively, *P* = 0.700), child’s dietary diversity was significantly higher in intervention areas (42.9% vs. 34.1%, *P* = 0.001). The practices of minimum acceptable diet was also significantly higher in intervention areas (31.2% vs. 25.3%, *P* = 0.017). The percentage of 6–23 months aged children ever fed or effectively fed MNP in last 6 months were significantly higher in intervention areas (16.2% vs. 7.4%, *P* < 0.001 and 3.76% vs. 0.9%, *P* = 0.001 respectively) (Table 3).

**Table 3:** Maternal practices on child feeding in comparison and intervention area

Characteristics	Comparison area	Intervention area	*P* ^2^
Number of children aged < 2 years (N)	769	974	
Prelacteal feeding (%)	13.26	9.75	0.022
Initiation of breastfeeding within 1 h of birth (%)	65.93	68.99	0.175
Number of children aged < 6 months (N)	133	256	
Exclusive breastfeeding (%)	59.40	72.66	0.008
Number of children aged 6–23 months (N)	612	699	
Initiation of complementary feeding by 7 month (%)	69.12	70.10	0.700
Having 4+ food groups out of 7 groups^1^ in last 24 h (%)	34.12	42.90	0.001
Minimum acceptable diet in last 24 h (%)	25.31	31.20	0.017
Ever fed multiple micro-nutrient powder in last 6 months (%)	7.39	16.16	< 0.001
Effectively fed multiple micro-nutrient powder in last 6 months (%)	0.94	3.76	0.001

^1^ 7 food groups: 1) grains, roots and tubers; 2) legumes and nuts; 3) dairy products (milk, yogurt, cheese); 4) flesh foods (meat, fish, poultry and liver/organ meats); 5) eggs; 6) vitamin-A rich fruits and vegetables; 7) other fruits and vegetables

^2^ *P*-value for the Chi-square test between comparison and intervention areas

### Nutritional status of under-5 children

The prevalence of stunting among under-5 children was significantly lower in intervention areas than comparison areas (28.8% vs. 37.2%, *P* < 0.001) (Table 4). After adjusting for potential confounders in the mixed effect logistic regression model, it was found that the children from intervention area had 25% lower odds of being stunted than those from comparison area (aOR: 0.75, 95% CI: 0.60–0.94, *P* = 0.012).

**Table 4:** Stunting among under-5 children in comparison and intervention area

Characteristics	Stunting	Unadjusted		Adjusted^a^	
	% (95% CI)	OR (95% CI)	*P*	OR (95% CI)	*P*
Area					
Comparison	37.19 (34.74–39.71)	1.00		1.00	
Intervention	28.77 (26.58–31.07)	0.69 (0.55–0.87)	0.002	0.75 (0.60–0.94)	0.012

^a^ OR adjusted with administrative zone, household wealth quintile, child’s age, gender, maternal age, education, occupation, cluster disparity and variation between study groups

## Discussion

The present study is an attempt towards assessing the role of a nutrition intervention in the form of nutrition counselling of mothers in reduction of stunting prevalence among under-5 children in Bangladesh. Nutrition counselling has often been considered as an effective tool for increasing maternal knowledge and practices on optimal child feeding. However, there is lack of evidence on rigorous assessment of the effectiveness of these initiatives in Bangladesh. The present study is one of the few studies carried out in Bangladesh assessing the effectiveness of nutrition counselling in attaining improved nutritional status among young children.

Overall, the study reported that the prevalence of stunting was significantly lower among children from intervention areas where mothers of these children received the nutrition counselling. We also found that nutrition counselling had a positive role on increasing some of the optimal IYCF practices, which might have resulted in significant reduction in stunting prevalence among children. Other researchers have pointed that attainment of optimal nutrition in early childhood is largely dependent on ensuring optimal IYCF practices [7, 35–37].

Optimal IYCF practices have been defined by the World Health Organization (WHO) as early initiation of breastfeeding within the first hour of delivery, exclusive breastfeeding for up to six months of life, introducing nutritionally rich complementary foods after six months of age and continued breastfeeding till two years of life [32]. We found that maternal practices on early initiation of breastfeeding (within one hour of birth), which is particularly crucial for neonatal survival [38], was significantly higher in intervention areas. Early initiation of breastfeeding also promotes prolonged period of exclusive breastfeeding [39], and it is recommended that children should be exclusively breastfed for 6 months of life to achieve proper growth and development [40]. The present study also reported that exclusive breastfeeding was significantly higher in the intervention areas. Furthermore, higher number of children from the intervention areas fulfilled minimum dietary diversity requirement of eating food items from at least 4 food groups, which is particularly important for the children to attain proper nutrition [41, 42]. A few studies [16, 17] carried out in similar settings also reported that counselling mothers on child feeding practices is associated with overall improvement in optimum infant and child feeding practices. Although, some studies found that maternal nutrition counselling results in reduced childhood stunting [19, 20], other studies [43, 44] reported that nutrition counselling were ineffective in reduction of stunting among young children. However, it was also reported through conducting a meta-analysis that nutrition education is effective in reducing stunting among young children when it is complemented with interventions such as food or micronutrient supplementation or nutrition safety net programs [45]. The positive correlation between maternal nutrition counselling and reduced stunting prevalence among young children in this study may be attributable to involving female community health workers and effective program monitoring.

The present study found that maternal knowledge on optimal child feeding practices was significantly higher in intervention areas compared to the comparison areas. The improvement in optimal child feeding practices in intervention areas might have been achieved due to this high level of maternal knowledge on child feeding practices. Studies carried out in similar settings also reported that maternal knowledge on child feeding is positively correlated with the child feeding practices and improved nutritional status among young children [46, 47]. The present research also pointed that face-to-face nutrition counselling to the mother is effective in increasing maternal knowledge as well as achieving proper IYCF practices among children. A previous study carried out in Dhaka, Bangladesh also reported that maternal nutrition education helps in motivating mothers to exclusively breastfed their children for up to 6 months [48]. Several other studies conducted in resource poor settings also pointed that maternal counselling on child feeding is effective for improving child feeding practices through increasing maternal knowledge on child feeding [14, 16, 49].

Bangladesh has a well-structured healthcare system with health facilities readily available. It has a setup of community clinics, one for each 6000 people, and CHWs are employed in the clinics with the role of community health service. They provide basic preventive and curative health services to the community, similar to that of the CHWs of BRAC EHC. However, with the goal of achieving strict targets on health outcomes, nutrition issues lose its importance on many occasions [7]. Therefore, to sustain the progress childhood stunting reduction and to accelerate the reduction rates, the government may initiate nutrition education to mothers through providing nutrition training to CHWs. At the same time, the government also needs to establish a strong monitoring system to ensure quality health and nutrition services at the community level.

The study result should be interpreted with caution as it was subjected to certain limitations. Firstly, no baseline information on childhood stunting was available, we were unable to rule out the possibility that the intervention children were less stunted at the outset. It is likely that not having these measurements before the intervention may result in a weak study design and therefore, a randomized controlled trial is recommended where the intervention group mothers receive the nutrition counselling by trained CHWs alongside the usual care while the comparison arm mothers receive the usual care. Second, one should interpret the results with caution considering differences in statistical and clinical significance. We caution the readers that they should pay attention to the actual differences between the groups as in some cases the differences were small to be meaningful even though the differences are statistically significant. Third, childhood stunting might be influenced by the parental biological factors which were not considered in the present study. Fourth, as the study follows a cross-sectional design, it may subjected to reporting bias. Finally, a number of factors such as repeated infections, worm infestation, and maternal hygiene and sanitation practices pertaining to the children was not recorded.

## Conclusions

The present study suggested that counselling mothers on child feeding practices may be effective in reducing the prevalence of stunting among under-five children. The finding is particularly crucial from policy perspectives, suggesting that provision of training on nutrition counselling to frontline community health workers can effectively motivate mothers to ensure proper feeding practices to their children.

## Data Availability

The datasets used and/or analyzed during the current study are available from the corresponding author on reasonable request.
